# Electroencephalography: electrode arrays in dogs

**DOI:** 10.3389/fvets.2024.1402546

**Published:** 2024-11-14

**Authors:** Stephen Everest, Luis Gaitero, Robert Dony, Alexander Zur Linden, Miguel A. Cortez, Fiona M. K. James

**Affiliations:** ^1^Department of Clinical Studies, Ontario Veterinary College Health Sciences Centre, University of Guelph, Guelph, ON, Canada; ^2^School of Engineering, College of Engineering and Physical Sciences, University of Guelph, Guelph, ON, Canada; ^3^Division of Neurology, Department of Paediatrics, Temerty Faculty of Medicine, University of Toronto; Neurosciences & Mental Health Program, Peter Gilgan Center Research Learning, SickKids Research Institute, Toronto, ON, Canada

**Keywords:** dogs, electroencephalography, epilepsy, seizures, standardized electrode placement

## Abstract

Electroencephalography (EEG) is the gold standard for confirming epileptic seizures in both human and veterinary patients. Despite idiopathic epilepsy being one of the most common neurological conditions in dogs, our understanding of it in veterinary medicine lags that in human medicine. The relative underuse of EEG in dogs with seizures has potential causes including practical issues, financial concerns, lack of training/equipment, and questions of clinical value. This technological underuse may lead to, or result from, major gaps in our understanding of EEG in veterinary patients. This underutilization of EEG is of significant clinical relevance because the diagnosis of specific epilepsy syndromes in humans guides the treatment, namely pharmacological, dietary, or surgical. These epilepsy syndromes are diagnosed based on several factors, one of which is the characteristic electrical brain activity on EEG. The aim of this narrative literature review was to highlight the study of cortical brain activity to improve our understanding of EEG in veterinary medicine. Specifically, the utility of EEG with focus on the existing proposed electrode arrays and their current supporting evidence. A recent survey study confirmed that a variety of canine EEG protocols are concurrently in use, including diverse electrode arrays. By comparison, in humans there is a standardized 10–20 electrode array, with average localization error of 13–17 mm depending on the number of placed electrodes on the scalp. We offer a review of the factors that would contribute to the ideal canine EEG electrode array highlighting areas for improvement and future validation. This proposed level of understanding will facilitate the identification of cortical seizure foci with a known degree of error, paving the way for non-pharmaceutical interventions like epilepsy surgeries.

## 1 Introduction

Electroencephalography (EEG) is essential for the characterization of epilepsies, seizure foci and specific epilepsy syndromes. This technique involves recording brain activity via electrodes placed on the scalp. In this way, EEG detects the transient abnormally synchronous cortical activity in the brain that manifests clinically as epileptic seizures (1, 2). In human medicine, EEG is the readily available diagnostic functional neurological tool to characterize epilepsy syndromes for specific treatments, i.e., antiseizure medications (ASMs) and, where appropriate, surgery to disconnect or remove the cortical epileptogenic focus. Each human epilepsy syndrome is defined based on etiology, seizure types, age of onset, imaging features, and EEG findings. In addition to specific treatment options, the diagnosis of an epilepsy syndrome provides prognostic implications (3). Epilepsy in dogs and people displays similar ictal and inter-ictal patterns on EEG (4). Yet in veterinary patients, such epilepsy syndromes have rarely been established, with most seizures in dogs being diagnosed based on clinical suspicion alone. Some breed-specific characteristics and epilepsy syndromes have been described. Further investigation into these, i.e., identification of pathogenic genetic variants, may ultimately facilitate breed specific treatment plans, similarly to treatment for human epilepsy syndromes (5). Veterinary patients may thus benefit from the clinical use of EEG similar to that in human medicine.

To strengthen the utilization of EEG in veterinary patients, the foundational weakness in our knowledge that must be overcome is that already presented by the great variety of canine EEG electrode arrays reported in the literature. This lack of consensus makes it impossible to reliably compare the EEG recordings between dog breeds and veterinary EEG centers. For people, an international standard 10–20 electrode placement array was established in 1958 by Jasper (6). In this array, electrodes are placed at 10 and 20% increments along lines measured between bony landmarks of the head, e.g., nasion to inion. Electrodes in the 10–20 system have an alphanumeric designation based on their location, where the letter indicates the cortical region under the electrode, e.g., ‘F’ for frontal lobe, with odd electrode numbers on the left, even electrode numbers on the right, and ‘z’ indicating midline scalp locations. Magnetic resonance imaging (MRI) has since confirmed consistent anatomical correlation to specific cortical regions, with reported average localization error of 17 mm when using a standard 21-electrode map in the 10–20 system array, and 13 mm when using a 41-electrode map for the 10–20 system array (7). Such replication studies not only validated the international standard for people but reinforced the use of the system for clinical comparisons between patients and its utility as the initial step for cortical source localization. In order to facilitate source localization, both the forward and inverse problems must be addressed. The forward problem is identifying the cortical source of the signal that is detected by the EEG electrodes whereas the inverse problem is identifying which electrodes would detect a signal from a given cortical source (8).

Besides nomenclature and location accuracy and precision, source localization is further supported by the number of electrodes in the array. For example, a positive correlation was determined between the number of electrodes used and the accuracy of source localization in pediatric patients (9). This determination was made by comparing the source localization of interictal spikes using 32, 64, 96 and 128 channel EEG recordings against surgical resection and intracranial recordings. Based on this paper, the International Federation of Clinical Neurophysiology (IFCN) recommended that EEG electrode arrays consisting of at least 64–76 electrodes should be used in people when attempting accurate source localization, with the extended 64-electrode array referred to as the 10–10% system (10, 11). This marks a notable shift toward the use of high-density EEG arrays in people containing 64–256 electrodes. This shift has been facilitated by technical advances and with devices such as expandable nets or caps to avoid the need for manual measurements. When not specifically attempting source localization, the IFCN recommends using a minimum of 25 electrodes when performing a standard EEG due to inadequate coverage of the temporal lobe when using fewer electrodes (12, 13).

In veterinary patients, various EEG arrays have been proposed since the 5-electrode array initially used by Redding and Knecht in 1984 (14–17). The veterinary use of nomenclature analogous to the 10–20 system began in the early 2000s (18). A consistent nomenclature reduces confusion and facilitates the use of translational models to further the understanding of EEGs in both human and veterinary species (18). Generally, veterinary electrode arrays have an unknown mapping of electrode location relative to cortical regions when placed using bony landmarks. Further unknowns include electrode location accuracy and precision, particularly with respect to agreement in inter- and intra-observer placement. Establishing a validated standard array with known localization errors will guide veterinary epileptology to take the next step toward defining epilepsy syndromes and also pursuing indicated epilepsy surgery. The aim of this narrative review, therefore, is to establish the level of the current understanding of EEG electrode arrays in veterinary patients, their current utilization in the profession, and limitations in our knowledge. Recognizing that arrays will need to be validated per species, the focus is on the EEG arrays proposed in dogs and their supporting evidence.

## 2 Indications for electroencephalography in canine epilepsy

Idiopathic epilepsy is one of the most common neurological brain condition in dogs, affecting as many as 0.6–0.75% of dogs in the general population and up to 9.5% in some breeds (19–21). It is a disease of great importance in veterinary medicine as epileptic patients are significantly more likely to be euthanized than those that are not, usually due to quality-of-life concerns (22). In veterinary medicine, the diagnosis of idiopathic epilepsy is documented by the International Veterinary Epilepsy Task Force (IVETF) according to the level of confidence within a 3-tier system. In this system, a tier-I level of diagnostic confidence is defined as a patient who is within the typical age range for onset of seizures in dogs with idiopathic epilepsy (6 months–6 years). They should also have experienced two or more seizures at least 24 h apart, unremarkable physical examination and inter-ictal neurological examination, and no identified cause for seizures on bloodwork or urinalysis. Tier II is achieved by the above attributes, with the addition that structural and metabolic causes for epilepsy are ruled out by performing a bile acid stimulation test, MRI and cerebrospinal fluid analysis. Finally, tier III confidence is established in patients who meet the above criteria, with EEG activity consistent with epileptic seizures. In this paradigm, the scalp EEG is the final confirmatory step, highlighting idiopathic epilepsy being a diagnosis of exclusion, in contract to human medicine where EEG is used earlier in conjunction with neuroimaging for the diagnosis.

When EEG is utilized early in the diagnostic process, it can help to differentiate between epileptic seizures and other non-epileptic paroxysmal events (17). Examples of non-epileptic paroxysmal events in dogs distinguished from epileptic seizures by EEG include fly-catching syndrome, in which EEG revealed spike activity in only 38% of the patients (23). Other indications for EEG use in veterinary practice include discrete event diagnosis, continuous state diagnosis, drug treatment monitoring, diagnosing brain death, type of seizure/epilepsy, epileptic seizure focus localization, sleep disorders and post-operative monitoring of brain surgery (24). The reliance on identifying outward manifestations of epileptic seizures for the diagnosis of epilepsy in dogs means that the underdiagnosis of both seizures and epilepsy in veterinary patients may be an ongoing clinical issue. A study by Packer et al. highlighted the unreliability of visual seizure diagnoses by observers (25). They invited 15 veterinary professionals, 10 of whom were neurology specialists, to complete a survey describing paroxysmal episodes depicted in 100 randomized videos of dogs or cats. Descriptions had to include whether the video showed an epileptic seizure or describe what the episode was if not an epileptic seizure. Responses were recorded, with percentage agreement and Fleiss’ kappa (*κ*) calculated for more than 2 observers for each variable in the questionnaire. Worryingly, there was only 29% inter-observer agreement, with a κ value of 0.4 (fair where a *κ* value of 0 = no agreement) on whether the animal in the video was experiencing an epileptic seizure. The highest level of agreement was for generalized tonic–clonic seizures (*κ* = 0.6) with the lowest agreement for focal seizures (κ = 0.31). This uncertainty among veterinary professionals is concerning given that seizures are usually witnessed by owners, the majority of whom are non-veterinary professionals. It is reasonable to assume the layperson would have less agreement on what a seizure looks like than the veterinarians who are trained to identify them. In supporting the idea that epilepsy is underdiagnosed in veterinary patients, this draws attention particularly to the underdiagnosis of focal or absence seizures. Absence seizures and focal seizures are hard to distinguish visually but arise differently in the brain cortical regions; absence seizures are generalized, whereas focal seizures are confined to one cerebral hemisphere (26). For this reason, EEG is the only way to distinguish between these two types of non-generalized tonic–clonic seizures. Seizure frequency may also be underestimated. The discrepancy between reported seizure frequency and true epileptic seizure frequency on EEG was confirmed by Ukai et al. where only a weak correlation was identified retrospectively (27). This suggests that EEG may be underutilized in veterinary patients, whether due to practicality issues, financial constraints, or questions of clinical need for it based on under-recognition of epileptic seizures. It also suggests that the seizure underreporting phenomenon described in humans may also exist in dogs and, along with it, associated consequences for patient care and accuracy of therapeutics trials (28).

The understanding of epilepsy and EEG in dogs continues to catch up to what is known in humans. While it has been confirmed that epileptic dogs show similar EEG patterns to human epileptic patients, epilepsy in dogs continues to be categorized broadly, with few and minimal descriptions of specific epilepsy syndromes (4). For people, the International League Against Epilepsy (ILAE) recognizes over 20 different epilepsy syndromes, with specific guidelines for treatment of each (29). They define these as epileptic disorders that are characterized by clusters of signs and symptoms that typically occur together. These include the type of epileptic seizure, etiology, anatomy, precipitating factors, age of onset, severity, chronicity, diurnal and circadian cycling, and sometimes prognosis (30). The eight epilepsy syndromes with EEG characterizations identified in dogs are mostly breed specific. These syndromes have been identified in Beagles, Belgian Shepherds, Cavalier King Charles Spaniels, Finnish Spitzs, Lagotto Romagnolos, Pomeranians, Rhodesian Ridgebacks and Standard Poodles, summarized in Table 1. These epilepsy syndromes in dogs are defined by (suspected) genetic background, EEG findings and seizure types; veterinary syndromes have yet to accrue the richness of detail associated with human syndromes. For example, juvenile epilepsy in Lagotto Romagnolos, characterized as a remitting benign epilepsy, is associated with an *LGI2* genetic variant (31). EEG findings reveal interictal sharp waves and spike waves, and the seizure types are either recurrent focal, or focal onset with secondary generalization (32). Seizures can also be classified based on semiology as proposed by the IVETF (33). The clinical manifestations described include motor, sensory/behavioral and autonomic. Motor manifestations involve skeletal musculature and can entail increased or decreased muscle contraction leading to movement. Sensory manifestations are subjective ictal phenomena that can include behavioral changes, e.g., fear, aggression, searching behavior or attention seeking. Finally, autonomic manifestations include any involvement of the autonomic nervous system. Typically, these signs would include ptyalism, mydriasis, urination or defecation.

**Table 1:** Summary of epilepsy syndromes recognized in dogs, characterized by breed, age of onset, EEG findings, and seizure type.

Breed/syndrome	Age of onset	EEG findings	Seizure semiology	Reference
Lafora disease in Beagles	Mean 8.3 years	Focal myoclonic polyspike-waves, interictal spikes, spike-waves, & polyspike-waves	Myoclonic episodes with/out generalized tonic–clonic seizures	Flegel et al., 2021; Demeny et al., 2020 (61, 62)
Idiopathic epilepsy in Belgian Shepherds	Mean 3.3 years	Interictal spikes and spike-waves, multiple foci	Focal onset with secondary generalization. Initial restlessness, attention seeking, ptyalism and nausea, followed by progression to stiffening of limbs and neck, muscle fasciculation, tremors, ptyalism, staring, falling, tonic–clonic convulsions and urination.	Seppala et al., 2012 (63)
Idiopathic epilepsy in Cavalier King Charles Spaniels	Not given	Interictal generalized spike-waves, ictal rhythmic sharp waves	Focal onset (majority) with/out secondary generalization. Repetitive fly catching, generalized tonic seizures or complex partial seizures	Driver et al., 2013 (64)
Idiopathic epilepsy in Finnish Spitzes	Median 3 years	Interictal focal or generalized spikes, polyspikes and spike-waves	Focal onset with secondary generalization	Vitmaa et al., 2006 (65) Jeserevics et al., 2007 (66) Vitmaa et al., 2013 (67)
Benign familian juvenile epilepsy in Lagotti Romagnoli	Mean 6.3 weeks	Focal interictal sharp waves and spike-waves	Focal or focal onset with secondary generalization	Jokinen et al., 2007 (31)
Neonatal encephalopathy with seizures in standard poodles	3–6 weeks	Frequent spikes, polyspikes and alpha-band rhythms.	Frequent spikes and polyspikes	Chen et al., 2008 (68) Yu et al., 2020 (69)
Idiopathic epilepsy and epilepsy of unknown cause in Pomeranians	Median 40.5 months	Focal or multifocal interictal spikes, sharp waves, polyspikes or a combination	Focal seizures, with limb contraction	Yu et al., 2022 (70)
Juvenile myoclonic epilepsy in Rhodesian Ridgebacks	Median 6 months	Ictal generalized 4 Hz spike-waves	Generalized absence with photosensitivity and myoclonus	Wielaender et al., 2015 (71)

The IVETF also categorizes idiopathic epilepsy into three groups based on etiology. These groups are genetic epilepsy, including dogs where a causative genetic variant or a confirmed genetic background is present; suspected genetic epilepsy, including dogs with a high breed prevalence (>2%) or familial accumulation of epileptic individuals; and finally, epilepsy of unknown cause, including patients for which there is no known genetic predisposition and no evidence of brain structural epilepsy related abnormality. In addition to the syndromes listed in Table 1, genetic epilepsy and suspected genetic epilepsy have been confirmed in certain breeds, comprehensively summarized by the IVETF in 2015 (5). Despite this burgeoning knowledge, there is yet to be consensus on treatment recommendations/responses to different ASMs in specific breed-specific epilepsies or syndromes. This is a major knowledge gap, resulting in selection of ASMs based on practicality and cost rather than matching them to the underlying etiology or epilepsy syndrome.

The prevalence of drug-resistant epilepsy in canine patients is a further concern in veterinary medicine. This is defined as inadequate seizure control despite pharmacological treatments with two or more indicated ASMs, at appropriate doses and with serum concentrations within the therapeutic range (34, 35). Drug resistant epilepsy is reported to affect as many as 20–40% of epileptic dogs, driving increasing interest in non-pharmaceutical treatment options like epilepsy surgery (34, 36). In order to facilitate epilepsy surgery, the precise source localization is required, highlighting the need to further our understanding and utilization of EEG in veterinary patients (37).

The future of veterinary epilepsy surgery was covered by Hasegawa, reviewing the concept of the epileptogenic zone in cats and dogs and summarizing our ability to detect it in veterinary patients (37, 38). This zone is defined as ‘the minimum amount of cortex that must be resected (or completely disconnected) surgically (39). It can be divided into 5 components, which can be challenging to identify when in different parts of the brain. These are the symptomatogenic, irritative, seizure-onset, and structurally abnormal zones (also known as an epileptogenic lesion). In humans, EEG in combination with video recording and magnetoencephalography (MEG), MRI and nuclear imaging can be used to identify these zones and facilitate epilepsy surgery. In veterinary patients with structural epilepsy, MRI is currently the most sensitive way to identify structurally abnormal epileptogenic zones. It is much more challenging in patients with idiopathic epilepsy due to the lack of gross structural changes to the brain. To determine the epileptogenic zone, multiple techniques including scalp EEG, invasive EEG, video EEG, functional MRIs and nuclear imaging are being studied in veterinary patients to determine their potential clinical/surgical utility (37). To support the localization of the epileptogenic zone, a clear understanding of the cortical anatomic correlates of scalp EEG electrode locations is needed.

Standardized scalp electrode locations would also benefit other electrophysiology modalities. Vanderzandt et al. demonstrated the ability to measure cortical somatosensory evoked potentials (SSEPs) in dogs following stimulation of the median and tibial nerves (40). They suggested that greater standardization of electrode placement when performing SSEPs in dogs could improve their consistency and help to distinguish SSEPs generated by physiological events from variations in technique (40). Their possible use has been suggested in humans with genetic generalized epilepsy, however similar findings have not yet been shown in dogs (41). Improved consistency when performing SSEPs would enable more reliable comparison between studies and further understanding of the effects of cerebral pathology on SSEPs, as in the case of epilepsy.

## 3 Veterinary clinical usage of electroencephalography

It is suspected that EEG in veterinary patients is underutilized, whether due to lack of understanding/training, equipment, practicality, or financial constraints. The first study reporting on EEG utilization in the profession was performed over 30 years ago (42). This was a small survey looking into the use of certain electrodiagnostics (EEG, spinal evoked potentials and brainstem auditory evoked potentials) in veterinary neurology. At that time, 34 questionnaires were sent to veterinary neurologists in North America, with 19 responses received. Of those, 17 reported that they were actively recording EEGs in dogs and cats. At that time, 14 of them were using the 5-electrode montage proposed by Redding and Knecht (14). Though a large majority of responders were performing EEGs, there was no indication of how many times per year they were performed. There was also a response rate of only 56%, which may not have been representative of the actual use of EEG, as veterinary neurologists that were performing them would likely have an interest in the survey and therefore responded to it. The information from that study is outdated, with multiple other electrode arrays proposed since it was published.

A more recent survey was performed by Luca et al. (24). This was a much larger study, with surveys sent via listserv to members of both the American College of Veterinary Internal Medicine Neurology section and the European College of Veterinary Neurology. Out of 400 invitations to participate, only 180 responses from veterinary neurologists worldwide were received. While the majority had performed EEGs in dogs at some point (75%), only 44% reported still using them. As suspected, the main reason these EEGs were performed was to determine whether dogs were truly having epileptic seizures. The primary reasons for not performing EEGs included lack of equipment, lack of training and experience, financial costs, and limited perceived diagnostic value. That less than half of veterinary neurologists reported using EEG confirmed that it is still relatively underutilized in veterinary medicine. This survey, however, only asked questions related to EEG in dogs, so current figures regarding EEG use in other veterinary species remain unclear. Based on the typical caseload in veterinary neurology practice though, it is reasonable to assume dogs are over-represented compared to other species. Additionally, a majority of responders reported only using EEG annually (70/119). Finally, electrode arrays appeared to be very variable between responders, with the number of electrodes ranging from 6 to 32. The most used array was the one proposed by Holliday and Williams, which was used by 31% of participants (43).

## 4 Electroencephalography electrode arrays in dogs

One of the major limitations of canine EEG is the need for a standardized, validated EEG electrode array. At the time of the survey performed by Steiss in 1988 (42) most veterinary neurologists performing EEG were using variations of the 5-electrode array proposed by Redding and Knecht in 1984 (14). This was a relatively rudimentary array which did not make use of the 10–20 electrode nomenclature already used in human EEGs. Their EEG array consisted of five recording, and a ground electrode. The electrodes were positioned over the frontal and occipital regions bilaterally, with one midline, vertex electrode. While this small number of electrodes helped to determine the presence of seizure activity, it left out large regions of the brain. By comparison, in human EEG, localization accuracy is demonstrably better when using more electrodes in 10–20 arrays, e.g., increasing from 21 to 41 electrodes improves localization error by 4 mm (7). This is important particularly when planning epilepsy surgeries for safe removal of the smallest amount of cortical tissue required to eliminate the seizure focus. Better localization would also help to characterize epilepsy syndromes by more accurately identifying the region of the brain the seizures are originating from.

**Figure 1:**
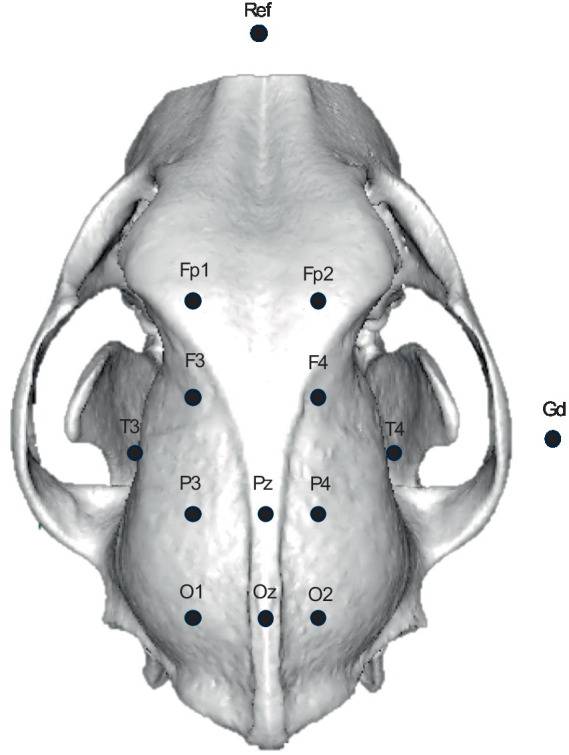
EEG array proposed by Pellegrino and Sica. The reference electrode (Ref) is positioned at midline on the most rostral part of the nasal bones and the ground electrode (Gd) is at the apex of the ear (16).

Pellegrino and Sica proposed a larger standardized EEG electrode array in 2004 (Figure 1) (16). This was a 12-electrode array, with instructions for electrode placement relative to established, palpable bony landmarks on the head such as the temporal crest, zygomatic arches, and mastoid processes, developed through a series of studies (44–50). The array used nomenclature analogous to the 10–20 array in human patients. To determine placement of the electrodes, the cadaveric heads of 80 dogs were examined, including 30 brachycephalics, 42 mesocephalics, and 8 dolichocephalics. Dissection of the dogs’ heads was performed after EEG electrode placement to confirm the anatomical position of the cerebral cortex relative to the electrodes, and the placement instructions were provided for each skull conformation type. This study, however, did not provide measures of the variance in electrode localization to cortical topography, a key first step to developing epilepsy surgery in dogs (37). To this end, replication of the Pellegrino and Sica array was presented as a conference abstract by Daniel et al. (51). Both manual dissection of one mesocephalic dog’s head and MRI neuronavigation were used to confirm location of electrode placement relative to the brain cortical regions. This was topographically represented over the cortical lobes. Both gross and virtual dissection methods had good agreement but found incomplete coverage of the frontal lobe while electrodes placed over the parietal lobe were caudally displaced. Conversely, replication with a single specimen was insufficient to measure either electrode placement error or clinician placement accuracy and precision. Going a step further, an abstract describing the use of MRI-guided neuronavigation to identify underlying cortical anatomy in a cadaver found that it facilitated more accurate placement of the EEG electrodes compared to using external skull topography, however this approach was not routinely practical (52). The facilitation by neuronavigation was confirmed in a larger study (53). Whether due to human error when placing the electrodes or a need for adjustments to the array map, further replications are needed to confirm complete brain coverage, quantify electrode placement error, and account for inter-clinician consistency. Such replication studies raise confidence in the reliability of findings and support the evidence base for scientific consensus as would be needed to create an acceptable standard EEG electrode array for dogs.

**Figure 2:**
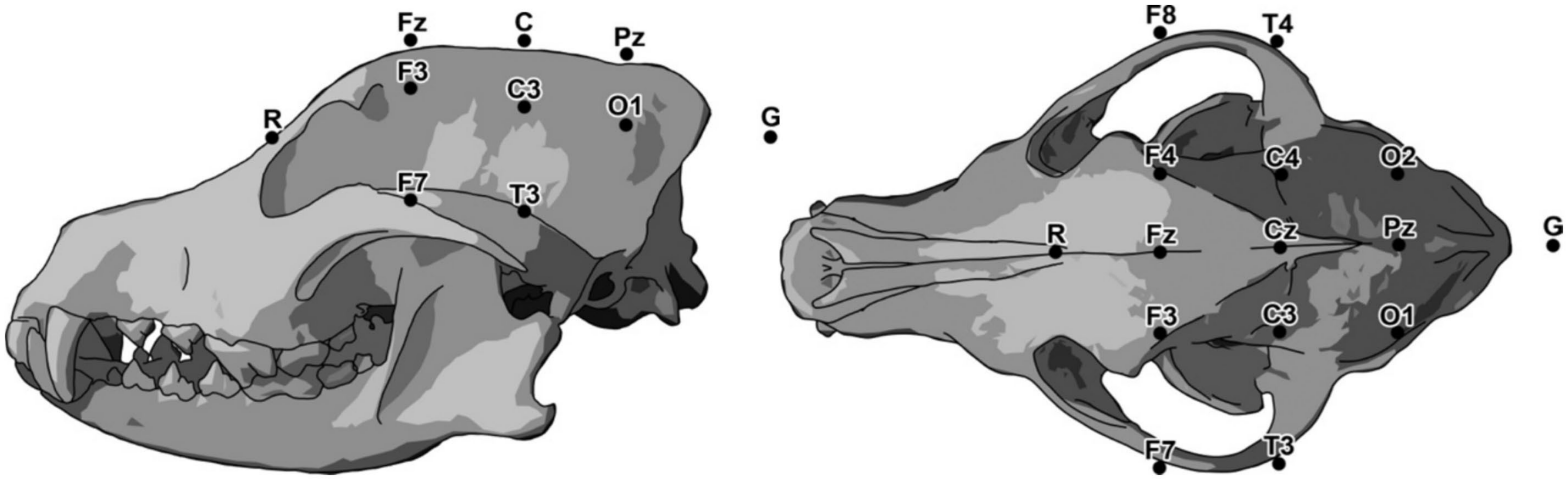
Modifications to the Pellegrino and Sica EEG array proposed by James et al. (17).

James et al. modified the Pellegrino and Sica array, incorporating a total of 15 electrodes in an attempt to achieve more cortical coverage and to move the reference and temporal electrodes to locations better tolerated by unsedated dogs (Figure 2) (16, 17). The previous array proposed by Pellegrino and Sica required placement of an electrode deep in the temporal muscle in close proximity to the skull in order to eliminate artifact originating from the temporal muscles. Sphenoid electrodes in human EEGs are similarly placed deep in the tissues of the head adjacent to the skull, though sphenoid electrodes are placed at the base of the skull different to the Pellegrino and Sica array. Sphenoid electrodes also differ from the Pellegrino and Sica array in the cortical source detected, anterior-inferomesial temporal lobe and pseudosylvian fissure, respectively (16). While sphenoid electrodes may provide more information in a small percentage of patients, they do not appear to be necessary in the majority of human epilepsy patients and are associated with pain (54). Cheek or anterior zygomatic electrodes can replace sphenoid electrodes in people with a small loss of spatial or temporal resolution (55). While the temporal to zygomatic electrode modification of the Pellegrino and Sica array may reduce discomfort for unsedated recordings in dogs, whether there is similar spatial or temporal data loss is not yet known.

While the array proposed by James et al. is aimed to improve cortical coverage, it still needs cerebrocortical topographic validation (24). That is, underlying cortical anatomy and consistency between patients whether due to skull conformation, inter-placer variation or clarity of instructions remains to be confirmed. Finally, quantification of electrode placement variability (localization error) remains to be performed, limiting the utility of the array regardless of the coverage achieved. Knowing the localization error would support future epilepsy surgery in dogs (37).

Aside from these electrode arrays, there have been numerous other arrays described in veterinary medicine, as outlined in Table 2. The major limitation of these arrays remains the lack of validation, i.e., known accuracy, precision, and localization error. Because of this, it is unknown which part of the brain each electrode is closest to or how reliably each electrode can be situated. This means source localization is currently not possible in veterinary patients. There is also significant variation of number of electrodes used in the diverse canine EEG arrays, with Luca et al. reporting a range of 6–32 (24). This is also highlighted in Table 2. As it has been established that, in humans, source localization can be performed with greater accuracy when using more electrodes, this suggests that arrays with fewer electrodes would likely have reduced accuracy or more significant gaps in their coverage than arrays with more electrodes (7). Conversely, EEGs with fewer electrodes still facilitate detection of epileptic cortical activity, and have the benefit of being quicker to place and likely better tolerated by veterinary patients. The use of fewer electrodes may also be of benefit in veterinary patients with smaller skulls to ensure correct electrode spacing (17). Finally, the number of electrodes may be limited by the number that the EEG device itself can accommodate. These points may be of benefit in situations where urgency is required, e.g., when using an EEG for status monitoring, or in non-epilepsy indications for EEG, e.g., sleep studies or polysomnography (56). Aside from the number of electrodes, it is notable that there has been a shift in the nomenclature used for the electrodes, with some of the earlier arrays (e.g., Holliday and Williams naming them based on their anatomic position), and more recent arrays, e.g., Bergamasco, Pellegrino and Sica and James et al., naming them based on similar electrodes from the human 10–20 arrays. This likely reflects awareness of and interest in comparative epilepsy investigations. With this in mind it should be noted that there are differences in brain and skull morphologies when comparing the anatomy of humans and dogs. For this reason direct transfer of the nomenclature used for the human 10–20 array to arrays used in dogs may not be the most appropriate solution, and may explain why studies by Holliday et al. and Utsugi et al. avoided the use of this terminology (57). For example, based on where the caudal midline electrode is placed, at the center of the occipital bone or more rostral, raises the question as to whether the electrode at this location should be called Pz or Oz. Ultimately, this would be decided by localizing the electrode to the underlying cortical topography, and testing the accuracy and precision of its placement. This uncertainty further highlights the need for consistent nomenclature and replication studies when considering veterinary EEG arrays and source localization. The morphological differences between species raise the question of whether the human 10–20 nomenclature should be used or whether one specifically for veterinary species should be formulated, although the latter option would make translational studies more complex.

**Table 2:** List of EEG arrays previously described in dogs.

Electrode array source	Number of electrodes (including ground and reference)	Montage channels	Electrode type	Sedation
Holliday et al. 1970 (59)	8	6 channels (LF, RF, LT, LP, RP, RT, LO, RO)	Subcutaneous	Pentobarbital, thiopental (doses not specified)
Redding and Knecht (1984) (14)	6	5 channels (LF, RF, V, LO, RO)	Not specified	Not specified
Jaggy and Bernadini 1998 (72)	10	8 channels, mono-, bipolar (LF, RF, V, LO, RO)	SNE, 12 mm	Medetomidine IV (0.025 mg/kg), propofol bolus IV (2 mg/kg), Propofol CRI (0.05–0.1 mg/kg/min), Atipamezole IV (0.125 mg/kg)
Berendt et al. 1999 (4)	16	14 channels (F3, F4, T3, C3, C4, T4, O1, O2)	Subcutaneous	Acepromazine, pethidine (doses not specified)
Holliday and Williams 1999 (43)	15	14 electrodes (S, LF, FV, RF, LT, LC, CV, RC, RT, LP, PV, RP, LO, RO)	Subcutaneous or surface electrodes	Mepiridine IM or SC (5 mg/kg), acepromazine IV (0.1 mg/kg)
Holliday and Williams 1999 (43) (used in very small patients)	13	12 electrodes (S, LF, FV, RF, LT, LC, CV, RC, RT, LP, PV, RP)	Subcutaneous or surface electrodes	Mepiridine IM or SC (5 mg/kg), acepromazine IV (0.1 mg/kg)
Morita et al. 2002 (73)	14	12 channels, mono-, bipolar (F1, F2, F3, F4, F5, P1, P2, T1, P3, T2, O1, O2)	Subcutaneous	Xylazine (dose not specified)
Bergamasco et al. 2003 (18)	19	17 channel ref. montage (F7, F3, Fz, F4, F8, T3, C3, Cz, C4, T4, T5, P3, Pz, P4, T6 O1, O2)	SNE, 15 mm	Propofol IV (6 mg/kg), propofol CRI (0.5–0.9 mg/kg/min)
Pellegrino and Sica 2004 (16)	14	12 electrodes (Fp1, Fp2, F3, F4, T3, T4, P3, Cz, P4, O1, Oz, O2)	SNE, 15 mm	Xylazine SC (1 mg/kg)
Brauer et al. 2011 (74)	7	5 electrodes (F3, F4, Cz, O1, O2)	SNE, 12 mm	Propofol IV (7.5 mg/kg), propofol CRI (0.37 mg/kg/min), rocuronium bromide IV (0.4 mg/kg)
Hasegawa 2016 (37)	15	13 electrodes (Fp1, Fp2, F3, Fz, F4, T3, C3, Cz, C4, T4, O1, Pz, O2)	Surface disk, SNE or SWE	Medetomidine 20–40 μg/kg, IM
Tepper and Shores 2014 (75)	6	5 electrodes (F3, F4, Cz, P3, P4)	SNE, 12 mm	Medetomidine IV (2 μg/kg)
Wrzosek et al. 2016 (23)	10	8 electrodes (F3, F4, C3, C4, T3, T4, O1, O2)	SNE or SWE	Not specified
James et al. 2017 (17)	15	13 electrodes, (F7, F3, Fz, F4, F8, T3, C3, Cz, C4, T4, O1, Pz, O2)	SNE or SWE	Mostly none, however dexmedetomidine, butorphanol, acepromazine and atipamezole were used as needed
Lyon et al. 2024 (60)	10	8 electrodes (FP1, FP2, T3, T4, C3, C4, O1, O2)	Surface cup or stud electrodes	Mostly none, however dexmedetomidine and/or butorphanol were used if needed

SNE, subdermal needle electrodes; SWE, subdermal wire electrode.

A variable that must be considered when performing EEGs in dogs is the marked variability in skull shape. The effect of skull configuration on EEG electrode localization relative to different regions of the brain is not yet fully understood. The effect of skull conformation on brain morphology was highlighted by Johnson et al. when trying to compose a stereotactic brain atlas (58). With respect to brachycephalic dogs, when assessing the Jacobian warping metric there was high levels of warping in the frontal and olfactory cortices. Based on this it was concluded that a specific brachycephalic population template was warranted based on the severity of the brain deformity associated with brachycephaly. Notably, the only EEG electrode array in dogs that attempts to account for brachycephaly is the one proposed by Pellegrino and Sica (16).

## 5 Discussion

In veterinary medicine, there is an opportunity to build a fundamental understanding of epilepsy and EEG in dogs. In human medicine, selection of ASMs and prognosis can be determined based on diagnosis of specific epilepsy syndromes (3). These syndromes are diagnosed based on age of onset, seizure focus, comorbidities, clinical characteristics, and brain electrical activity on EEG. In comparison, veterinary patients are classified using a rudimentary system based on clinical manifestations, essentially grouping idiopathic epileptics into genetic/suspected genetic versus epilepsy of unknown cause, with unproven genetic involvement (33). This is not ideal, as the blanket treatment/trial and error approach that neurologists are reliant upon may be resulting in inappropriate ASM selection in some cases.

With regards to EEG in dogs, the 2023 survey by Luca et al. highlighted how underutilized EEG is in the veterinary profession, with fewer than 50% of responders currently performing EEG (24). This survey showed significant improvement compared to the one performed by Steiss in 1988 with almost 10 times as many responses, and was balanced by the possibility that the number of responses may be biased as those responding may have had more interest in EEG research than those that did not (43). While lack of training and equipment are often listed as some of the primary reasons for not performing EEG, in the more recent survey, 58.82% of those performing EEGs were only doing so annually. This suggests that even when appropriate equipment and training are present, EEG remains underutilized. This could be due to financial concerns or perceived lack of clinical value, further demonstrating the need to encourage performance of EEGs in veterinary patients with suspected seizures. The large range in number of electrodes used in arrays (6–32 electrodes), as well as the fact that the most used array is only used by 31% of responders highlights the lack of consensus and the need for a standardized electrode array for comparison between veterinary EEG centers.

Consensus on a standardized electrode array would be foundational for veterinary epileptology. Without it, the ability to identify seizure foci is limited, which in turn can prohibit the identification of certain epilepsy syndromes as well as performing epilepsy surgeries in patients refractory to medical management. The purpose of a standardized array would be to facilitate accurate electrode placement with adequate coverage achieved in the majority of patients. If inconsistent coverage is due to user error, this would imply that more specific instructions are required to remove the inter-user error between patients. Though MRI neuronavigation achieved more accurate electrode placement for the Pellegrino and Sica array, this is impractical in most instances and would limit EEG electrode placement to patients who had already had an MRI performed. An imaging-based standardized array would be predicted to have poor uptake due to the need for expensive advanced imaging to perform a significantly cheaper test. The ideal electrode array would be non- or minimally-invasive to allow flexibility for use in patients with or without sedation. The ideal clinical array would be easily reproducible, with known error, fulsome cortical coverage, and easily identified landmarks when placing the electrodes. The first step toward this ideal array will be to validate existing arrays like the Pellegrino and Sica original or modified arrays, using both palpable bony landmarks and neuroimaging (16). The objective would be to confirm coverage over the frontal and parietal lobes and indicate where adjustments should be made to this array to achieve more accurate and consistent coverage (51). If it is not possible to ensure an adequate number of electrodes (i.e., in patients with smaller skulls), it would be beneficial to establish core electrodes, which should be placed following from Holliday and William’s initial suggestion while also ensuring functional topographic correlates with the human 10–20 system for optimal translational comparisons (59). This may be made easier by using a neuronavigation approach as suggested by Rogers et al. but may ultimately be determined by solving the inverse problem of source localization for dogs (53). There have been no published attempts to validate any other existing electrode arrays, presenting a major gap in our knowledge and limiting our ability to both diagnose and treat epilepsy in veterinary patients.

When attempting to develop a standardized EEG electrode array, significant factors in dogs are the variability in skull conformation across breeds, as well as variability in skin elasticity and mobility. This is something that has not been quantified in dogs or cats but is visibly much more of an issue than in people, where scalps are relatively immobile. This presents veterinary specific challenges when developing an array, as skin elasticity and mobility could influence electrode placement even by changing patient position. This means that regardless of inter-operator electrode placement accuracy, the same electrode could detect signals from different parts of the brain during the same recording due to patient movement. One solution for managing skull and skin variables would be the development of an EEG cap to hold the electrode. Such a cap would also control for inter- and intra-operator electrode placement errors, ultimately a seemingly simple solution. Demonstrating convergence of thought, a recent similar approach used a custom headset and later a commercial elastic strap cap to maintain EEG electrodes in place (60). This study demonstrated the feasibility of such devices, with the next step being to assess the consistency of electrode localization to the cortex using this method. While that study is a promising step in the development of a standardized EEG cap, further investigation is required to determine the localization error of electrode placement with this method.

In conclusion, there are currently many gaps in our EEG protocols for dogs. Of particular importance is the development of a standardized electrode placement array, to improve our ability to reliably identify the clinically suspected epileptic seizure localization. The validation and adoption of a standardized electrode array would pave the way for the identification of epileptic syndromes and also facilitate the indication of epilepsy surgeries in veterinary patients.
